# The processing of inter-item relations as a moderating factor of retrieval-induced forgetting

**DOI:** 10.2478/v10053-008-0117-x

**Published:** 2012-08-21

**Authors:** Tobias Tempel, Werner Wippich

**Affiliations:** Department of Psychology, University of Trier, Germany

**Keywords:** retrieval-induced forgetting, generation effect, episodic memory, recall, inhibition

## Abstract

We investigated influences of item generation and emotional valence on retrieval-induced forgetting. Drawing on postulates of the three-factor theory of generation effects, generation tasks differentially affecting the processing of inter-item relations were applied. Whereas retrieval-induced forgetting of freely generated items was moderated by the emotional valence as well as retrieval-induced forgetting of read items, even though in the reverse direction (Experiment 1), fragment completion eliminated the moderation of retrieval-induced forgetting by emotional valence (Experiment 2). The results corroborate the assumption that the processing of inter-item relations is crucial for the immunization against retrieval-induced forgetting. Moreover, differential processing of inter-item relations may clarify the mixed results on moderating factors of retrieval-induced forgetting that have been reported.

## Introduction

Memory for generated items is mostly better than memory for read items (Bertsch, Pesta, Wiscott, & McDaniel, 2007). Generation effects have been very thoroughly investigated. Up to the present time, it remains an open question, however, as to how retrieval affects previously generated items as opposed to read items.

One recently well-examined consequence of retrieval is the phenomenon of retrieval-induced forgetting (RIF), in which the selective retrieval of a subset of items causes the forgetting of the non-retrieved subset. RIF typically is investigated with the retrieval-practice paradigm (Anderson, Bjork, & Bjork, 1994) that consists of three main phases. In the encoding phase, participants study several sets of items in combination with a shared cue that defines the specific set. The material mostly used are exemplars of semantic categories like *mumps* and *influenza* that share the category cue *illness*. In the retrieval-practice phase, participants are then cued to recall half of the studied exemplars from half of the categories by completing a series of cued-stem tests (e.g., *illness-inf*…). In the test phase, they are finally cued with each category name and asked to recall any of the exemplars from the encoding phase. Recall performance is assessed on three types of exemplars: practiced exemplars from practiced categories (Rp+ items), unpracticed exemplars from practiced categories (Rp- items), and unpracticed exemplars from unpracticed categories (Nrp items). RIF manifests itself in significantly lower recall of Rp- items compared to Nrp items.

Although RIF is a robust phenomenon, it does not always occur. With regard to boundary conditions of RIF, one can ask (at least) three questions: What is immunized against RIF, why is it immunized, and how is it immunized?

Examining the first question, one stimulus quality has been given special attention in previous investigations. Several studies found differences in RIF depending on the emotional valence of the material (Dehli & Brennen, 2009; Harris, Sharman, Barnier, & Moulds, 2010; Iglesias-Parro & Gomez-Ariza, 2006; Kuhbandner, Bäuml, & Stiedl, 2009; Wessel & Hauer, 2006). Emotional items obviously can be immunized against RIF: Dehli and Brennen (2009), for example, found RIF only for exemplars of neutral categories, but not for exemplars of positive or negative categories. The literature on the role of valence is somewhat inconsistent, though. Whereas in some studies RIF did not occur for emotional stimuli (e.g., Dehli & Brennen, 2009; Iglesias-Parro & Gomez-Ariza, 2006), in others it did (Amir, Coles, Brigidi, & Foa, 2001; Storm, Bjork, & Bjork, 2005). Studies using autobiographical memories as items are especially interesting in this context because among these are the only studies that demonstrated immunizations against RIF for positive, but not for negative, material. Whereas a study by Barnier, Hung, and Conway (2004) found RIF of neutral, positive, and negative autobiographical memories, Wessel and Hauer (2006), as well as Harris et al. (2010), reported experiments in which no forgetting for positive, but only for negative autobiographical memories, occurred.

Why do immunizations against RIF occur for emotional items (sometimes)? Possibly, the self-relevance of the material defines its susceptibility to RIF and thus accounts for different moderations of RIF by valence for passively encoded items and autobiographical memories generated individually by participants. Perhaps positive autobiographical memories are spontaneously encoded in a way that prevents forgetting, whereas negative memories are not likewise protected. For this reason, the participants’ September 11 memories in a study by Coman, Manier, and Hirst (2009) also might not have been immunized against RIF. In contrast, emotional items that are not generated by the participant, perhaps, generally tend to be encoded in a way that prevents forgetting because emotionality usually indicates which stimuli are worth being attended to. Put differently, when items are generated - that is, being retrieved from memory - negative items might be prone to be easily forgotten in a later test and hence become subject to RIF. When predetermined stimuli have to be encoded, on the other hand, emotional items in general might tend to be immunized against RIF because any emotional information in the environment potentially is of high importance to the perceiver.

The repeated demonstrations of immunizations against RIF for emotional material prove that this stimulus quality is an important influence, albeit occurrences of RIF for emotional material indicate that it is not a highly robust influence. Examining emotional intensity as the potential source of variations in the occurrence of RIF, Kuhbandner et al. (2009) found that RIF occurred for negative stimuli of low intensity, but higher emotional intensity was associated with a decrease in RIF. Low emotional intensity thus might explain the emergence of RIF of negatively valenced items in some studies (Amir, Coles, Brigidi, & Foa, 2001; Storm et al., 2005). However, a different cause could be that no immunizing processing quality was triggered; that is, emotional valence indeed determines which items potentially get immunized against RIF, but whether they in fact are immunized is determined by their specific processing. Other factors besides valence should be able to influence the particular quality by which items are processed.

To explain how immunizations against RIF occur, two encoding processes have been proposed: distinctiveness and inter-item integration. Hunt (2006) defined *distinctiveness* as the processing of difference in the context of similarity. Smith and Hunt (2000) induced distinctiveness in that sense by instructing participants to state differences between to-be-learned exemplars of a semantic category. No RIF occurred for such distinctively encoded items. The concept of distinctiveness also has already been used to explain moderations of RIF by the valence of the stimulus material. Kuhbandner et al. (2009) argue that encoding items in terms of differences between the items accounts for the immunizations of emotional material against RIF that have been reported so far. In a similar vein, Dewhurst and Parry (2000) provided evidence that memory advantages for emotional words over neutral words can be attributed to the distinctive encoding of emotional words. However, immunizations against RIF for emotional items could alternatively be explained by integrative encoding. Perhaps the usually higher semantic relatedness of emotional items compared to neutral items (Talmi & Moscovitch, 2004) makes emotional items more susceptible to integration. *Integration* refers to the explicit formation of connections between the items of a study list. Anderson and McCulloch (1999) manipulated integration by instructing participants either only to memorize exemplars of several categories or, while proceeding in their learning, to remember all previously presented exemplars of a category each time a new exemplar of that category was presented. The latter instruction caused an immunization against RIF. Anderson and McCulloch also found that participants who indicated they had been engaging in spontaneous integrative rehearsal during encoding did not show RIF, whereas those participants who reported no or only little integrative rehearsal did.

Manipulating factors that affect distinctiveness and integration should influence the occurrence of RIF, depending on valence. We assume that, even though the two processes differ, distinctiveness and integration both depend on the processing of inter-item relations. On the one hand, inter-item relations enable the comparison of features when differences between items are processed. On the other hand, inter-item relations are building blocks of item integration. We used generation tasks to influence the processing of inter-item relations.

According to the three-factor account of generation effects (McDaniel, Waddill, & Einstein, 1988; Steffens & Erdfelder, 1998), item generation can influence the processing of inter-item relations either positively or negatively. Alongside item-specific processing (the first factor) and the processing of item-cue relations (the second factor), the processing of inter-item relations constitutes the third factor within this theoretical model. *Item-specific processing* refers to the depth and elaboration of the processing of a single item. The processing of item-cue relations determines how strongly a generated item is connected to the cue its generation depended on. In addition, levels of processing also determine item-cue connections. If the generation task is conceptual, the item-cue connection will be primarily conceptual as well. If the generation task, on the contrary, is perceptual, the item-cue connection will be primarily perceptual. Lastly, the processing of inter-item relations determines how strongly and on which level items of one list are connected with each other. In a list of exemplars of one category, for example, items can be connected conceptually as all having the same relation to the category name, whereas in a list of words all beginning with the same letter, items can be interrelated perceptually. Whenever organized item lists are encoded (as is the case within the retrieval-practice paradigm), the third factor comes into play. Generation can either enhance or diminish the processing of inter-item relations.

The three-factor account of generation effects assumes that generation always enhances item-specific processing, leading to a memory advantage of generated items over read items in recognition tests. Generation is also posited to enhance the processing of item-cue relations. For example, generating *mumps* from the cue *illness* produces a stronger association of the two words than reading the word pair. Consequently, cued-recall tests reliably yield memory advantages for generated items as well. The third factor (the processing of inter-item relations) is assumed only to benefit from generation, though, when inter-item relations are congruent to item-cue relations. However, generation prevents the processing of inter-item relations when inter-item relations are incongruent to item-cue relations. Congruence exists when items have to be generated in response to the same cue. Incongruence exists when each item of an organized list is generated in response to a different cue. For instance, the conceptual inter-item relations of exemplars of one semantic category are congruent to the name of the category but not to individual phonological rhyme cues or perceptual cues. The conceptual inter-item relations of the items *mumps* and *influenza* would be congruent to the category name *illness*, but incongruent to phonological cues like *pumps* and *bonanza* or perceptual cues like word fragments, for example *mu…ps* and *in…luenza*. Item generation is a problem-solving task (cf. Jacoby, 1978). The solution consists of finding the correct response to a given cue. Therefore, the item-cue relation is of primary importance. Diverting attention to relations between items that differ qualitatively from the item-cue relation would hinder the success of generation. Hence, processing of incongruent inter-item relations is prevented. Given the task of filling in the missing letters in *mu…ps* and *in…luenza*, it would not only be unnecessary to consider the common membership of mumps and influenza in the category of illnesses, but actually would not help solving the fragments. Free recall is especially sensitive to the processing of inter-item relations. Thus, a memory advantage for generated items emerges in a free recall test if the processing of inter-item relations is enhanced because of their congruency to item-cue relations, but a disadvantage (i.e., a negative generation effect; Schmidt & Cherry, 1989) emerges in the case of incongruency.

When a generation task promotes the processing of inter-item relations because of a congruency of item-cue relations and inter-item relations, then generated items might be immunized against RIF. In the case of incongruency, however, no immunization should occur. In addition, a promotion or prevention of processing of inter-item relations by item generation should affect the moderation of RIF by stimulus qualities such as emotional valence. Assuming that differential integration or distinctiveness mediate the effects of emotional valence, a generation task promoting the processing of inter-item relations, therefore, should be able to diminish any moderation due to a general immunization against RIF, whereas a generation task preventing the processing of inter-item relations should diminish any moderation, because neither integration nor distinctiveness should arise. We thus expected RIF of emotional and neutral items to differ between a reading condition and a generation condition. Taking into account previous research on generation effects for emotional items, we assumed though that generation would similarly affect emotional and neutral baseline items (Nrp items). Berrin-Wasserman, Winnick, and Borod (2003) examined effects of item generation and emotional valence in one experiment. Generation enhanced memory for both neutral and emotional words in a similar fashion.

Drawing on postulates of the three-factor theory of generation effects, we tested the prediction that item generation would affect RIF. We included emotional valence as an independent variable to examine the influence of generation on the moderation of RIF by this stimulus quality. Based on the assumption that the processing of inter-item relations accounts for immunization against RIF, we employed gene-ration tasks that would either promote (Experiment 1) or prevent (Experiment 2) the processing of inter-item relations.

## Experiment 1

In Experiment 1, we used the retrieval-practice paradigm, but modified the encoding phase by letting participants freely generate exemplars in response to category names as the sole cues in one experimental condition. Four negative and four neutral categories were used. In the retrieval-practice phase, half of the exemplars from two negative and two neutral categories had to be retrieved. Subsequently, recall for all items was assessed. To promote the processing of inter-item relations in order to intensify a potential moderation of RIF by emotional valence, the items were presented in blocked order. In the reading condition, all exemplars of one category were presented at the same time, and in the free-generation condition, all exemplars of one category had to be generated consecutively. Assuming that influences of emotional valence were mediated by integration or distinctiveness, in the reading condition, we therefore expected less RIF of exemplars of negative categories than of neutral categories or no RIF of exemplars of negative categories at all (cf. Dehli & Brennen, 2009). In the free-generation condition, we expected a general immunization against RIF independent of emotional valence due to the additional promotion of processing of inter-item relations by the act of generating several exemplars in response to the same category name. However, the special material of individually generated exemplars could also suggest the alternative expectation that RIF would emerge for negative but not for neutral categories. RIF of generated exemplars might be influenced by the valence of the category in a parallel way as RIF of autobiographical memories. Perhaps generating autobiographical memories as items and generating the more abstract material of exemplars of semantic categories have in common that the generated material is highly self-relevant. Negative (but not neutral or positive) material, then, might be prone to forgetting.

### Method

#### Participants, design, and materials

A total of 80 psychology students at the University of Trier, 61 women and 19 men, participated in the experiment. They received course credits for their participation. Three variables were manipulated:Encoding conditions (reading, free generation) were manipulated between subjects. Emotional valence (negative, neutral) and item type (Rp+, Nrp, Rp-) were manipulated within subjects.

The stimuli were 10 category names and 60 exemplars, six exemplars from each category. The eight experimental and two filler categories (*planet* and *cereal*) were drawn from two published German norms (Mannhaupt, 1983; Scheithe & Bäuml, 1995). The categories were matched on the mean number of associations produced in the respective norm studies. Four of the experimental categories were neutral (*source of light, flower, predator*, and *object of art*) and four were negative (*illness, criminal, natural disaster*, and *vermin*).

Six exemplars from each category were selected for the reading condition. These exemplars had a rank order between 1 and 10. Their three initial letters were unique among all used items. In a pilot study, nine participants rated the emotional valence of the category names and the to-be-read exemplars on a 7-point scale ranging from -3 (*very negative*) to 3 (*very positive*). The average rating for negative category names was -2.3 (*SD* = 0.6), and the average rating for neutral category names was 1.1 (*SD* = 0.5). The average rating for exemplars of negative categories was -1.8 (*SD* = 0.4), and the average rating for exemplars of neutral categories was 0.8 (*SD* = 0.3). So, neutral categories were rated as slightly positive.

#### Procedure

The experiment consisted of three main phases separated by distractor tasks. In the encoding phase, participants were randomly assigned to one of two tasks. Each participant was given a stack of sheets with the instructions for the first task printed on the first page. Participants were instructed either to read lists of category exemplars, or to freely generate exemplars in response to category names. The time to read the exemplars of one category was limited to 20 s, after which a computer controlled sound signal indicated the need to turn to the next page, that is, the next category. The free-generation task was not time limited, so participants proceeded through the categories at self-determined speeds, turning to the next page after having written down six exemplars. In the reading condition, each page had a category name printed at the top and six exemplars underneath it. In the free generation condition, only a category name was printed at the top of the page. The first and the last category were the two filler categories. The experimental categories were presented as alternating negative and neutral categories. In both conditions participants were instructed to memorize the exemplars for a later test.

After the encoding phase, participants performed a first distractor task: 5 min of algebra. In the subsequent retrieval-practice phase, participants were given another stack of sheets. On the first page they were instructed that the next pages contained category names together with word stems of previously studied exemplars, and that they should recall the demanded items and complete the word stems. The next page contained the three-letter word stems of four items from the filler categories, each additionally cued with the corresponding category name (e.g., *cereal-bar*…). The subsequent pages then each contained word stems of items of the four to-be-retrieved-practiced categories (e.g., *illness-inf*…). The order of presentation was randomized. After all the word stems of the Rp+ items had been presented, they were presented again in the same fashion, but in a different random order. So, every Rp+ item had to be retrieved twice. The repeated retrieval of the Rp+ items is a usual procedure intended to ensure the occurrence of RIF. Then four word stems of four items from the filler categories were presented. The participants proceeded through this task at individually determined speeds. In the reading condition, we counterbalanced which categories were practiced and, also, which exemplars within a category were practiced. This process yielded four retrieval-practice sets. In the free-generation condition, the experimenter compiled the individual sets for the subsequent retrieval-practice during the preceding distractor task. He selected half of the exemplars from half of the categories the participant had generated, typed the three initial letters of the selected exemplars into a personal computer in order to create the retrieval-practice set, and finally printed the set. We counter-balanced which categories were practiced. In addition, for half of the participants, the items generated at the first, fourth, and sixth position were selected, for the other half, the items generated at the second, third, and fifth position were selected.

After a second distractor task - 3 min of algebra - the test phase began. Participants were given a third stack of sheets. On the first page they were instructed that every subsequent page contained a category name and that they should write down all exemplars they could recall from the encoding phase in 30 s. The next page contained the name of the filler category planet. Then the names of the experimental categories were presented in a random order.

### Results and discussion

#### Encoding phase and retrieval-practice phase

Some of the freely generated items could not be regarded as correct exemplars of the given categories. The most frequent among those items were famous person names and artworks (e.g., *Al Capone* or *The Scream*). Altogether 6.9% of the generated items were incorrect, even though conceptually related to the respective category. No completely unrelated items were generated. Therefore, no items were excluded from further analyses. In addition, we computed the overlap between the freely generated items and the 10 most common exemplars of the respective categories (Mannhaupt, 1983; Scheithe & Bäuml, 1995). The items of negative categories overlapped to 49.2%. The items of neutral categories overlapped to 62.9%.

Retrieval success in the retrieval-practice phase was very high and did not differ significantly between the two encoding conditions (reading: 96.2%; free generation: 96.4%; *t* < 1).

#### Test phase

Recall rates in the test phase are shown in Table 1. To examine the enhancement of Rp+ items we conducted a 2 (encoding condition: reading, free generation) × 2 (emotional valence: negative, neutral) × 2 (item type: Rp+, Nrp) ANOVA with repeated measures on the second and third factor. The main effect of item type was significant, *F*(1, 78) = 77.86, *p* < .001. Rp+ items profited from the retrieval-practice. The Item Type × Encoding Condition interaction was significant too, *F*(1, 78) = 20.37, *p* < .001. A ceiling effect probably was responsible for lesser enhancement of Rp+ items in the free-generation condition. The other interactions were not significant (*F*s < 3.20, *p*s ≥ .08).

**Table 1.** Experiment 1: Mean Proportion of Items Recalled as a Function of Item Type, Encoding Condition, and Emotional Valence.

	Reading						Free generation					
	Rp+		Nrp		Rp-		Rp+		Nrp		Rp-	
	*M*	*SE*	*M*	*SE*	*M*	*SE*	*M*	*SE*	*M*	*SE*	*M*	*SE*
Negative	.85	.03	.61	.03	.55	.04	.92	.02	.85	.02	.75	.03
Neutral	.80	.03	.63	.04	.50	.04	.94	.02	.88	.02	.84	.03
Total	.83	.02	.62	.03	.53	.04	.93	.01	.86	.01	.80	.02

*Note*. Rp+ = practiced exemplars from practiced categories. Nrp = unpracticed exemplars from unpracticed categories. Rp- = unpracticed exemplars from practiced categories.

To examine RIF we conducted another 2 (encoding condition: reading, free generation) × 2 (emotional valence: negative, neutral) × 2 (item type: Nrp, Rp-) ANOVA with repeated measures on the second and third factor. The analysis showed a significant main effect of encoding condition, *F*(1, 78) = 59.62, *p* < .001. More freely gene-rated than read items were recalled. The main effect of item type was significant too, indicating RIF, *F*(1, 78) = 21.50, *p* < .001. The main effect of emotional valence was marginally significant, *F*(1, 78) = 3.40, *p* = .07, indicating a slightly better recall performance for neutral items. Whereas the Item Type × Encoding Condition and Item Type × Emotional Valence interactions were not significant (*F*s < 1), the interaction of Encoding Condition and Emotional Valence, *F*(1, 78) = 7.88,*p* < .01, as well as the three-way interaction, *F*(1, 78) = 4.18, *p* < .05, were significant. Additional t-tests revealed that performance for neutral Rp- items was reliably lower than performance for neutral Nrp items only in the reading condition, *t*(39) = 3.75, *p* < .001 (two-tailed), but not in the free-generation condition, *t*(39) = 1.23, *p* = .23 (two-tailed). Performance for negative Rp- items, on the other hand, was reliably lower than performance for negative Nrp items only in the free-generation condition, *t*(39) = 2.99, *p* < .01 (two-tailed), but not in the reading condition, *t*(39) = 1.48, *p* = .15 (two-tailed).

It is known that the first items that are produced in a recall task can interfere with the retrieval of related items (Roediger & Schmidt, 1980). Lower recall of Rp- Items compared to Nrp items might reflect such output interference instead of RIF. Perhaps, participants initially retrieved the highly accessible Rp+ items. This might have interfered with the retrieval of the less accessible Rp- items. The difference in recall performance between Rp- items and Nrp items should be the larger, the earlier Rp+ items were retrieved. To investigate potential contributions of output interference, a difference score was calculated for each participant by subtracting the average recall position of Rp+ items from the average recall position of Rp- items (cf. Macrae & MacLeod, 1999). Then, a median split on these scores constituted an early Rp+ group and a late Rp+ group in each encoding condition, and their respective forgetting effects were compared, calculated as the difference in recall performance for Nrp items and Rp- items. With regard to the previously reported reliable forgetting effects, the early Rp+ groups did not produce larger forgetting effects than the late Rp+ groups, but, in fact, the forgetting effects were slightly smaller (read exemplars of neutral categories: .13 vs. .14; freely generated exemplars of negative categories: .08 vs. .12).

In summary, free generation did not immunize against RIF. It is possible that the generation task did not promote the processing of inter-item relations - or at least not as strongly as expected. However, the processing of inter-item relations probably was not prevented by generation either. Presumably, the different item material in the two conditions exerted stronger influences than differences in the processing of inter-item relations.

A partial immunization against RIF emerged in both encoding conditions, even though in opposing directions. The idiosyncrasy of the generated items may account for an opposing susceptibility to inter-item integration or distinctiveness, depending on the emotional valence. In a way, the generated items might have resembled the autobiographical memories used as the item material in the studies of Wessel and Hauer (2006) and of Harris et al. (2010) in which no forgetting for positive, but only for negative autobiographical memories occurred. Autobiographical memories and the more abstract material of exemplars of semantic categories in our study both have to be generated individually and differ from participant to participant. Such freely generated items are perhaps generally highly self-relevant, whereas preselected items are not, and negative self-relevant information may be more prone to forgetting than neutral and positive information. If this is true, using a generation task that requires the generation of exactly the same items as in the reading condition should disentangle the influences of generation and the influences of differential item material.

## Experiment 2

In Experiment 2, we investigated influences of fragment completion on RIF using identical items as in the reading condition of Experiment 1. Since free generation can be considered as the clearest operationalization of a generation task establishing congruent item-cue and inter-item relations, and our main interest was to examine the influences of item generation on RIF, we decided to use a generation task that would establish the incongruency of item-cue and inter-item relations. Using fragment completion also allowed us to control the time spent on generation. In contrast to the non-time-limited free generation in Experiment 1, potential differences in encoding time between the categories thus could not exert any influence.

In fragment completion of several exemplars of one category, item-cue and inter-item relations are incongruent because the success of filling in one missing letter in word fragments does not depend on the exemplars belonging to the same category. Instead, it mainly depends on the word fragment itself as the relevant cue, whereas the category name is of secondary relevance. For example, exemplars of the category *illness*, like *mumps* and *cancer*, are irrelevant for filling in the *n* in *influe…za*. The relation of the to-be-generated item to its cue, the word fragment, is per se perceptual because it consists of the sole missing letter. The conceptual inter-item relations are irrelevant and may even hinder successful generation if attention is paid to them. Their processing, therefore, is prevented. Accordingly, we expected not to find any immunization against RIF. Exemplars of negative and neutral categories should be forgotten.

## Method

A total of 40 psychology students at the University of Trier, 31 women and 9 men, participated in the experiment. They received course credits for their participation. Two variables, emotional valence (negative, neutral) and item type (Rp+, Nrp, Rp-), were manipulated within subjects. The stimuli were the same 10 category names and 60 exemplars used in the reading condition of Experiment 1.

The experiment again consisted of three main phases separated by distractor tasks. In the encoding phase, each participant was given a stack of sheets with the instructions for the first task printed on the first page. Participants were instructed to solve presented fragments of category exemplars by inserting one missing letter. The time to solve the fragments of one category was limited to 20 s. On each page a category name was printed at the top of the page and underneath fragments of six exemplars. The items were presented in the identical order as in the reading condition of Experiment 1. Participants were instructed to memorize the exemplars for a later test. The rest of the experiment corresponded exactly to the reading condition of Experiment 1.

## Results and discussion

### Encoding phase and retrieval-practice phase

The participants correctly completed 99.2% of the fragments. The remaining (not or incorrectly completed) items were excluded from all further analyses. Retrieval success in the retrieval-practice phase was 98.1%.

### Test phase

Recall rates in the test phase are shown in Table 2. To examine the enhancement of Rp+ items we conducted a 2 (emotional valence: negative, neutral) × 2 (item type: Rp+, Nrp) ANOVA with repeated measures on both factors. The main effect of item type was significant, *F*(1, 39) = 185.58, *p* < .001. Rp+ items profited from the retrieval practice. The main effect of emotional valence was not significant, *F*(1, 39) = 2.93,*p* = .10, neither was the interaction, *F*(1, 39) = 3.30, *p* = .08.

**Table 2.** Experiment 2: Mean Proportion of Items Recalled as a Function of Item Type and Emotional Valence.

	Rp+		Nrp		Rp-	
	*M*	*SE*	*M*	*SE*	*M*	*SE*
Negative	.79	.03	.53	.02	.43	.04
Neutral	.88	.02	.54	.03	.44	.04
Total	.83	.02	.53	.02	.43	.03

To examine RIF we conducted another 2 (emotional valence: negative, neutral) × 2 (item type: Nrp, Rp-) ANOVA with repeated measures on both factors. The significant main effect of item type indicated RIF, *F*(1, 39) = 10.47, *p* < .01. The main effect of emotional valence and the interaction both were not significant (*F*s < 1).

The investigation of potential contributions of output interference showed that the early Rp+ group again did not produce larger forgetting effects than the late Rp+ group (neutral categories: .08 vs. .10; negative categories: .10 vs. .12).

We also compared the recall performance for Nrp items with the recall performance for Nrp items in the reading condition of Experiment 1. Significantly fewer Nrp items were recalled in Experiment 2, *t*(78) = 2.34, *p* < .05 (two-tailed). This negative generation effect indicates that the processing of inter-item relations, in fact, was prevented.

In summary, no immunization against RIF emerged. Fragment completion apparently prevented the processing of inter-item relations. Accordingly, exemplars of negative categories just as exemplars of neutral categories could not be integrated, neither could they be processed distinctively.

## General Discussion

RIF of generated items differed from RIF of read items. The aim of our investigation was to test predictions derived from the three-factor theory of generation effects (McDaniel, Waddill, & Einstein, 1988; Steffens & Erdfelder, 1998). On the one hand, based on the assumption that the processing of inter-item relations accounted for immunization against RIF, we expected to find a general immunization for items generated in a task promoting the processing of inter-item relations or the emergence of RIF only for negative but not for neutral items. The latter result would parallel studies that demonstrated RIF for negative but not for positive autobiographical memories (Harris et al., 2010; Wessel & Hauer, 2006). The self-relevance of freely generated material in general might determine that negative material is forgotten easily, but neutral and positive material is not. On the other hand, no immunization for items generated in a task preventing the processing of inter-item relations was expected.

Freely generated items were not generally immunized against RIF. The emotional valence moderated forgetting for freely generated and for read items (Experiment 1), but not for items initially completed from word fragments (Experiment 2).

The immunization against RIF of read exemplars of negative categories corresponds with previous investigations (e.g., Dehli & Brennen, 2009). The results for freely generated items, on the other hand, resemble the results obtained by Wessel and Hauer (2006) and by Harris et al. (2010). These studies reported exclusive immunizations of positively valenced material accompanied by RIF of negatively valenced material, using autobiographical memories as items that naturally had to be generated individually by participants. Selective retrieval may shape individual associations to abstract concepts according to a similar principle as it shapes personal memories. The act of generating autobiographical memories and generating freely more abstract items perhaps both trigger processes that immunize neutral and positive information against forgetting, but not negative information. The function of such a partial immunization against forgetting might lie in the higher self-relevance of neutral and positive information (cf. Macrae & Roseveare, 2002). In the task of free generation used here, participants spontaneously named the first instances that popped into consciousness. Perhaps a high degree of self-relevance characterized these associations. Negative information then might be forgotten more easily as opposed to neutral information, due to some self-serving purpose. Analogically, the research on self-reference effects in memory has consistently shown that in normal subjects self-referentially processed neutral or positive information is better retained than negative information (e.g., Kuiper & Derry, 1982).

We consider the prevention from processing of inter-item relations in Experiment 2 as accounting for the absence of a difference in forgetting for items of negative and neutral categories there. Of course, this assumption needs further empirical support. Future studies might seek to identify other properties that are associated with differential forgetting and then to investigate if those differences do not occur following an initial task that prevents the processing of inter-item relations, just as fragment completion did here. Besides, the mixed results concerning the role of emotional valence for RIF perhaps can be seen not only to be due to differences in emotional intensity (Kuhbandner et al., 2009), but also to to the congruency, or respectively, incongruency of item-cue and inter-item relations. Storm et al. (2005), for instance, did not find a moderation of RIF by emotional valence. They used names of people as cues and trait adjectives as items. The inter-item relations in this study, therefore, can be regarded as incongruent to the item-cue relations, that is, relating a trait to an arbitrary name does not concur with relating different traits of the same valence. Hence, no moderation by emotional valence could emerge.

It was not within the scope of our study to differentiate between distinctiveness and integration as accounting for immunizations against RIF. Instead, the processing of inter-item relations, arguably being the common basis of both processing qualities, was manipulated. With regard to integration, the importance of inter-item relations should be apparent. The better the processing of inter-item relations, the better can items be integratively connected. If, however, distinctive encoding caused the partial immunizations against RIF, the results demonstrated a dependence of distinctiveness on inter-item relations that seldom has been taken into consideration in the investigation of distinctiveness. Indeed, distinctiveness is often equated with item-specific processing. Hunt (2006), in contrast, elucidates the importance of defining *distinctiveness* as the processing of difference in the context of similarity by highlighting the necessity of operating with precise concepts when researching memory and of relinquishing redundant terms in order to avoid imprecise theoretical models and circularity. Our investigation concurs with this notion. The three-factor account of generation effects represents a model that provides sophisticated assumptions about the co-action of three different encoding processes. In the present context, the three-factor account possibly needs to be even further elaborated by differentiating the third factor, the processing of inter-item relations, into integrative versus distinctive encoding. In any case, the application of this model to the phenomenon of RIF and its moderation by emotional valence showed that the third factor is of crucial importance regarding the answering of the question as to how retrieval affects generated as opposed to read information.
